# Epigenetic control of the ubiquitin carboxyl terminal hydrolase 1 in renal cell carcinoma

**DOI:** 10.1186/1479-5876-7-90

**Published:** 2009-10-26

**Authors:** Barbara Seliger, Diana Handke, Elisabeth Schabel, Juergen Bukur, Rudolf Lichtenfels, Reinhard Dammann

**Affiliations:** 1 Martin Luther University Halle-Wittenberg, Institute of Medical Immunology, Halle, Germany; 2 Martin Luther University Halle-Wittenberg, AWG Tumour Genetics of the Medical Faculty, Halle, Germany

## Abstract

**Background:**

The ubiquitin carboxyl-terminal hydrolase 1 (UCHL1) gene involved in the regulation of cellular ubiquitin levels plays an important role in different cellular processes including cell growth and differentiation. Aberrant expression of UCHL1 has been found in a number of human solid tumors including renal cell carcinoma (RCC). In RCC, UCHL1 overexpression is associated with tumor progression and an altered von Hippel Lindau gene expression.

**Methods:**

To determine the underlying mechanisms for the heterogeneous UCHL1 expression pattern in RCC the UCHL1 promoter DNA methylation status was determined in 17 RCC cell lines as well as in 32 RCC lesions and corresponding tumor adjacent kidney epithelium using combined bisulfite restriction analysis as well as bisulfite DNA sequencing.

**Results:**

UCHL1 expression was found in all 32 tumor adjacent kidney epithelium samples. However, the lack of or reduced UCHL1 mRNA and/or protein expression was detected in 13/32 RCC biopsies and 7/17 RCC cell lines and due to either a total or partial methylation of the UCHL1 promoter DNA. Upon 2'-deoxy-5-azacytidine treatment an induction of UCHL1 mRNA and protein expression was found in 9/17 RCC cell lines, which was linked to the demethylation degree of the UCHL1 promoter DNA.

**Conclusion:**

Promoter hypermethylation represents a mechanism for the silencing of the UCHL1 gene expression in RCC and supports the concept of an epigenetic control for the expression of UCHL1 during disease progression.

## Background

The highly conserved ubiquitin-proteasome complex is in addition to its general function in the protein turnover process also associated with the regulation of cell growth, differentiation, the modulation of membrane receptors and cellular stress responses as well as the turnover of different cytoskeletal components. It is comprised of enzymes involved in the protein ubiquitination/deubiquitination as well as of the subunits of the 20S proteasome that degrades ubiquitin-conjugated proteins [1,2]. Ubiquitination is a reversible biological process consisting of enzymes, that attach single or multiple ubiquitin molecules to protein substrates and deubiquinating enzymes (DUB), e.g. ubiquitin carboxyl-terminal hydrolases (UCH) and ubiquitin- specific proteases (USP) [3,4]. The protein gene product 9.5 (PGP 9.5) also termed ubiquitin carboxyl-terminal hydrolase-1 (UCHL1), a member of the UCH protein family, represents a soluble 25 kD protein with both ubiquitin hydrolase and dimerization-dependent ubiquitin ligase activities [5,6]. As a member of the ubiquitin-proteasome complex UCHL1 is involved in the control of the intracellular proteolysis, protein turnover and regulatory processes, which are important in maintaining normal cellular homeostasis [7]. UCHL1 expression exhibits marked tissue specificity and is mainly expressed in testis and neuronal tissues at various differentiation stages [8,9]. In addition, UCHL1 expression was detected during kidney development, in particular during the differentiation of renal tubules representing the origin of clear cell renal cell carcinoma (RCC) and in the regulation of the cell cycle of parietal epithelial cells of the Bowman's capsule [10,11]. Since UCHL1 is expressed in pathophysiological situations of the kidney such as acute ischaemic renal failure, renal hypertrophy, von Hippel Lindau (VHL) disease as well as neoplastic transformation of renal cells it may play a fundamental role in the mechanisms controlling the protein turnover of the kidney. There exists conflicting evidence concerning the role of UCHL1 in tumorigenesis varying from anti-tumor to pro-tumor properties depending on the tumor type analysed [12-14]. Several studies demonstrated aberrant UCHL1 expression in acute lymphoblastic leukaemia, myeloma, melanoma, neuroblastoma, pancreatic, esophageal, lung, thyroid, colon and renal cell carcinoma (RCC). In certain tumor types UCHL1 expression is even associated with tumor progression and decreased survival rates of patients [12,13,15-21]. However there is also evidence that UCHL1 expression might be associated with suppression of tumor growth in RCC [21]

DNA methylation at CpG dinucleotides within the promoter region of genes is a common event in the pathogenesis of tumors including urological cancers and has been explored as both mechanism and marker of tumor progression with potential application for diagnosis, classification and prognosis of disease [22-29]. Using different technologies UCHL1 has been identified as a frequently silenced gene in a cancer-specific manner, in particular in pancreatic, gastric, colon, ovarian, head neck squamous cell and hepatocellular carcinoma [14,30-35]. Thus, in order to understand the underlying molecular mechanism of the aberrant UCHL1 expression in RCC lesions [21], microarray analysis of the RCC cell line ACHN either left untreated or treated with the demethylating agent 2'-deoxy-5-azacytidine (DAC) was performed demonstrating an aberrant hypermethylation of the UCHL1 promoter DNA and an association with UCHL1 downregulation in RCC lesions [36]. We here extended these data and determined whether the promoter DNA methylation also contributes to the lack of UCHL1 expression in 32 pairs of primary RCC lesions and corresponding tumor adjacent kidney epithelium as well as 17 RCC cell lines. The given methylation status of the UCHL1 promoter DNA was further correlated with the UCHL1 mRNA and protein expression levels in these samples. Moreover, silenced UCHL1 expression could be restored in RCC cell lines by treatment with the demethylating agent DAC.

## Methods

### Cell lines and tissue culture

The human RCC cell lines employed in this study were established from patients with primary RCC of the clear cell type [21,37,38]. All tumor cell lines were maintained in high glucose Dulbecco's modified Eagles medium (DMEM) supplemented with 10% fetal calf serum, 2 mM glutamine, 100 U/ml penicillin/streptomycin, 1 mM non-essential amino acids and 1 mM sodium pyruvate (Gibco/BRL, Life Technologies, Karlsruhe, Germany).

### Patients and tumor biopsies

This study used tumor specimens of RCC obtained from patients undergoing nephrectomy at the Department of Urology of the University Hospital in Mainz, Germany. All cases had been reviewed by a pathologist according to the WHO classification criteria. Clinicopathologic data obtained from the patients included sex, age, TNM stage and histological subtype. The study design was approved by the Ethical committee of the Johannes Gutenberg University of Mainz and informed consent was obtained from all RCC patients.

### DAC treatment

To assess the ability of the DNA methyltransferase inhibitor DAC to induce the expression of UCHL1, RCC cell lines were treated for 5 days with 1, 5 and 10 μM DAC (Sigma-Aldrich GmbH, Taufkirchen, Germany). Subsequently untreated and DAC treated cells were harvested, lysed and total mRNA and/or total protein extracted. The resulting samples were then subjected to qRT-PCR, Western blot and methylation assays.

### Semi-quantitative and real-time reverse transcription polymerase chain reaction ((q)RT-PCR) analysis

Total RNA was extracted from the samples using the RNeasy Mini Kit (Qiagen, Hilden, Germany) according to the manufacturer's instructions. cDNA was synthesized from 3 μg RNA treated with DNase I (Invitrogen GmbH, Karlsruhe, Germany) using oligo dT primers (Fermentas, Mannheim, Germany) and Superscript II reverse transcriptase (Invitrogen). Real time PCR was performed with the UCHL1-specific primer set (sense: 5'-GCCAATGTCGGGTAGATG-3'; anti-sense: 5'-AGCGGACTTCTCCTTGTC-3') using an annealing temperature of 62°C. β-actin served as the reference gene (sense: 5'-GAAGCATTTGCGGTGGACGAT-3'; anti-sense: 5'-TCCTGTGGCATCCACGAAACT-3'. All real time PCR analyses were performed in a thermal cycler (Rotorgene, Corbett Life Science, Australia) using the QuantiTect SYBR-Green PCR Kit (Qiagen). UCHL1 expression levels were normalized against β-actin amplicons. The UCHL1 expression after 5-days DAC treatment was calculated as x-fold expression of the respective untreated sample, which was set to 1.

### Western blot analysis

20 μg of total protein/lane from untreated or DAC-treated RCC cell lines was subjected to Western blot analysis as previously described [21]. The membranes were incubated either with the anti-UCHL1-specific polyclonal rabbit antibody (PG 9500, BIOMOL, Hamburg, Germany) or with the anti-β-actin-specific monoclonal antibody (mAb) AC15 (ab6276, Abcam Ltd., Cambridge, UK) serving as a loading control. Horseradish peroxidase (HRP)-conjugated swine anti-rabbit IgG (P0217, DAKO, Hamburg, Germany) or rabbit anti-mouse IgG (P0260, DAKO) were used as secondary antibodies. The immunostaining was visualized using a chemiluminescence detection kit (LumiLight Western Blotting Substrate, ROCHE Diagnostics GmbH, Mannheim, Germany) according to the manufacturer's instructions.

### DNA extraction and analysis of the methylation status of the UCHL1 promoter

In order to investigate the methylation status of the UCHL1 promoter DNA, a CpG islet within the UCHL1 promoter containing 22 CpG dinucleotides was mapped using the CpGplot tool (EBI Tools, EMBOSS CpGPlot; ). Subsequently, bisulfite-specific primers flanking the transcription start site of the CpG islet in the UCHL1 promoter were designed with the Oligo 4.0 program relying on the reference sequence GI: 16949651 (National Bioscience, MN, USA). Upon isolation of genomic DNA from established RCC cell lines and/or biopsy specimens with the QIAamp DNA Mini Kit (Qiagen), 1 μg of DNA sample was subjected to bisulfite modification as previously described [39]. The methylation status of the UCHL1 promoter was determined using combined bisulfite restriction analysis (COBRA) as well as sequencing [39]. Briefly, 100 ng bisulfite treated DNA was amplified in 25 μl reaction buffer containing 0.2 mM dNTP mix, 1.5 mM MgCl_2_, 2 U Taq polymerase and 10 pmol of the primers 5'-GAG TTT TAG AGT AAT TGG GAT GGT GAA-A-3' and 5'-CCA CTC ACT TTA TTC AAC ATC TAA AAA ACA-3' using the following conditions: denaturation at 95°C for 3 min and 20 sec, primer annealing at 56°C for 25 seconds (25×) and primer extension at 72°C for 40 seconds and 5 min. The resulting amplicon (536 bp) was subjected to a nested PCR amplification with a set of internal primers (sense: 5'-GGT TTT GTT TTT GTT TTT TTT GTA TAG GTT-3' and antisense: 5'-AAA AAC AAA TAC AAA AAA AAA AAC AAA ACC-3') using 1/5^th ^of the first PCR product using the same PCR conditions, but extended to 30 cycles. Subsequently, 20-50 ng of the resulting PCR products (265 bp) were digested with 10 U BstU I and Taq I (New England Biolabs, Beverly, MA, USA) prior to separation on 2% Tris-acetate EDTA agarose gels.

For bisulfite genomic sequencing, the PCR products were gel-purified employing the PCR Purification Kit (Qiagen) according to the manufacturer's instructions and thereafter directly subjected to sequence analysis by a commercially available service provider (MWG Biotech, Martinsried, Germany). To analyse single sequences the purified PCR products were cloned into the pCR II vector using the TOPO TA Cloning Kit (Invitrogen) and subsequently the inserts of individual colonies subjected to sequence analysis.

## Results

### Correlation of the UCHL1 expression level in RCC cell lines of the clear cell type with the promoter DNA methylation status

**Figure 1:**
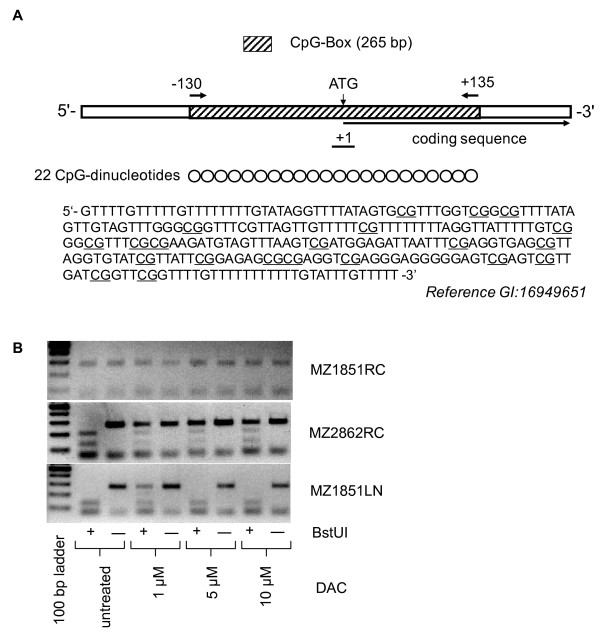
**UCHL1 promoter in RCC cell lines**. A) Schematic diagram of the UCHL1 core promoter DNA region with its respective CpG islet. The sequence segment of interest taken from the reference GI 16949651 as indicated is displayed below the scheme. The putative methylation sites (CpG dinucleotides) are underlined in the sequence stretch. (B) Representative COBRA pattern for RCC cell lines displaying a distinct methylation status of the UCHL1 promoter DNA (MZ1851RC: unmethylated; MZ1851LN: fully methylated; MZ2862RC: partially methylated) are shown. Genomic DNA extracted from the given RCC cell lines upon treatment with different DAC concentrations was treated with bisulfite and amplified by nested PCR as described in Methods. The resulting 265 bp amplicons were either digested with BstU I (+) or left untreated (-) and subsequently separated in 2% agarose gels in TAE buffer. A 100 base pair DNA ruler loaded in the first lane served as length standard.

We have recently demonstrated a heterogeneous expression pattern of UCHL1 mRNA and/or protein in both RCC cell lines and RCC lesions, which is associated with the RCC subtype, VHL status and with tumor progression [21]. In order to investigate the molecular mechanism(s) involved in this heterogeneous expression pattern, the DNA methylation status of the CpG islet in the UCHL1 promoter was determined in a series of 17 established primary RCC cell lines exhibiting heterogeneous UCHL1 expression levels. As determined by RT-PCR and Western blot analysis, 3/17 RCC cell lines express neither UCHL1 mRNA nor protein, 4/17 RCC cell lines exhibit low UCHL1 transcription, but no UCHL1 protein, whereas 9/17 express high levels of UCHL1 mRNA and protein (Table 1; [21]). Based on this screening we tested whether the lack of UCHL1 expression in RCC cell lines could be attributed to aberrant CpG islet methylation within its promoter region, which represents a common mechanism of gene silencing in various human cancers [31,34]. Therefore, the DNA methylation status of a genomic 265 bp DNA fragment containing 22 CpG dinucleotides next to the transcriptional start site of the UCHL1 gene (Figure 1A) was investigated by both COBRA and direct bisulfite sequencing. As representatively shown for 3 RCC cell lines in Figure 1B, the methylation pattern of the UCHL1 promoter DNA was highly heterogeneous varying from total to partial to lack of methylation. In MZ1851RC cells for example the UCHL1 promoter DNA was not methylated, whereas the COBRA-based analysis indicated a partial methylation of the UCHL1 promoter DNA in the RCC cell line MZ2862RC, characterized by methylation of some of the CpG dinucleotides within the core region of the UCHL1 promoter while other CpG sites remain unmethylated. In addition strong methylation of the promoter DNA core region, as defined by either methylation of all CpG sites or only few unmethylated CpG sites within the core region of the CpG islet, was found in the RCC cell line MZ1851LN. The status of the methylation pattern was directly associated with the response to DAC treatment: RCC cell lines with a strongly methylated UCHL1 promoter DNA responded to low concentrations of DAC (1 μM, MZ1851RC), whereas higher DAC doses were required to efficiently demethylate partially methylated promoters (10 μM, MZ2862RC). Based on the methylation status RCC cell lines could be classified into 3 different subgroups. The first category consists of RCC cell lines with a high to complete UCHL1 promoter DNA methylation predominantly lacking both UCHL1 mRNA and protein expression. The second exhibits a partially methylated promoter, which corresponds to low to moderate UCHL1 expression levels, whereas the third category is represented by RCC cell lines with unmethylated promoters expressing high levels of UCHL1 (Table 1). In order to verify the COBRA results and to determine the extent of methylation bisulfite DNA sequencing of the respective UCHL1 promoter region was performed in representative RCC cell lines [see Additional file 1]. As summarized in Table 1, the bisulfite DNA sequencing data confirmed the heterogeneous methylation pattern of the UCHL1 promoter detected by COBRA in RCC cell lines, but also stressed the point that there exists no strict homogeneity in regard to the methylation status of CpG oligonucleotides. Even within a given cell line the efficacy of the DAC treatment varied from the demethylation of 1 to 18 CpG dinucleotides within the UCHL1 promoter DNA (data not shown). Nevertheless, the data suggest that UCHL1 hypermethylation is tightly associated with the transcriptional silencing of UCHL1 in RCC cell lines.

**Table 1:** Association of the UCHL1 mRNA and protein expression pattern with the methylation status

	**UCHL1 expression**		**Methylation pattern**		
**RCC cell line**	**mRNA**	**protein**	**BstU I**	**Taq I**	**sequencing**

MZ1257RC	+	+	U	U	U

MZ1774RC	+	+	U	U	U

MZ1790RC	(+)	-	M	M	P

MZ1851RC	+	+	U	U	U

MZ1851LN*	(+)	-	M	M	M

MZ1879RC	-	-	M	M	M

MZ1940RC	-	-	M	M	M

MZ1973RC	+	+	U	U	U

MZ2175RC	-	-	P	P	P

MZ2733RC	+	+	U	U	U

MZ2789RC	+	-	P	P	P

MZ2858RC	+	+	U	U	U

MZ2861RC	+	+	U	U	U

MZ2862RC	(+)	-	P	M	P

MZ2885RC	+	n.d.	U	U	U

MZ2904RC	+	+ (pp)	P	P	P

MZ2905RC	+	+	U	U	U

*Cell line derived from a lymph node metastasis of a patient suffering from RCC. The methylation pattern of the UCHL1 promoter DNA was determined by COBRA and/or sequencing.

(-): no expression detectable; ((+)) weak expression detectable; (+) expression detectable; (U) unmethylated UCHL1 promoter; (P): partially methylated UCHL1 promoter (M): fully methylated UCHL1 promoter; (pp): expression verified by proteomic profiling of the corresponding RCC lesion; n.d. not done

### Restoration of UCHL1 expression in RCC by treatment with DAC

**Figure 2:**
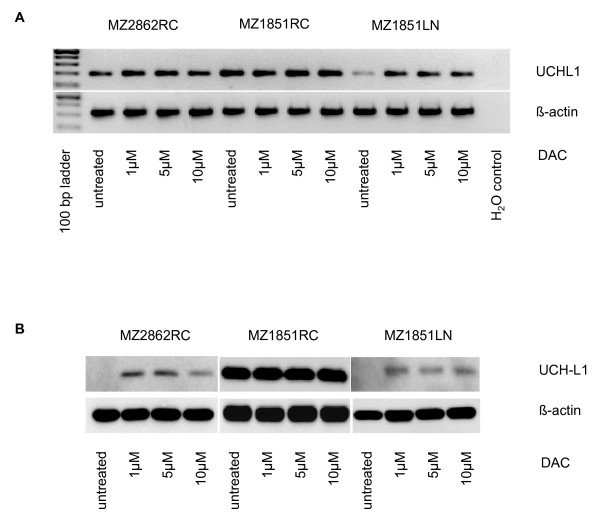
**Restoration of UCHL1 expression by DAC treatment in RCC cell lines**. The representative RCC cell lines either left untreated or treated with 1, 5, 10 μM DAC for 5 days were subjected to UCHL1-specific semi-quantitative RT-PCR (A) and Western blot analyses (B) as described in the Methods section.

To confirm that UCHL1 promoter DNA hypermethylation is responsible for the silencing of UCHL1, a selected number of UCHL1^- ^and UCHL1^+ ^RCC cell lines were treated with different concentrations of DAC (1, 5, 10 μM) for 5 days. As shown in Figure 2, DAC treatment of RCC cell lines displaying either partially (MZ2862RC) or fully methylated (MZ1851LN) UCHL promoter DNA regions led to the induction of UCHL1 mRNA (Figure 2A) restoring protein expression (Figure 2B). However, as representatively shown for MZ1851RC in RCC cell lines lacking UCHL1 promoter DNA methylation DAC treatment did neither alter the mRNA nor the protein expression levels of UCHL1. In contrast, the restored UCHL1 expression was associated with a partial or total demethylation of the UCHL1 promoter DNA as determined by COBRA (Figures 2A and 2B). Based on qRT-PCR analyses the induction at the mRNA level ranges from 1.1 - 1.4 fold in the RCC cell line MZ1851RC (unmethylated UCHL1 promoter DNA) to 11 - 13 fold in the RCC cell line MZ1851LN (strong methylated UCHL1 promoter DNA) to 11 - 18 fold in the RCC cell line MZ2862RC (partially methylated UCHL1 promoter DNA).

### Methylation of UCHL1 in human primary RCC lesions, but not of corresponding normal kidney epithelium

**Figure 3:**
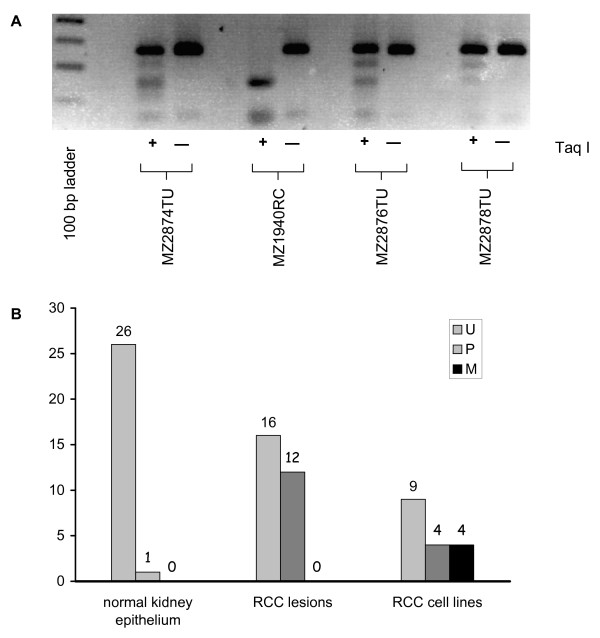
**UCHL1 promoter DNA methylation in RCC lesions, tumor adjacent kidney epithelium and RCC cell lines**. A) Representative COBRA analysis of three RCC tumor lesions and one RCC cell line. Genomic DNA extracted from tumor lesions (2874TU, 2876TU and 2878) and the cell line MZ1940RC was treated with bisulfite and amplified by nested PCR as described in the Methods section. The resulting 265 bp amplicons were either digested with Taq I (+) or left untreated (-) and subsequently separated in 2% agarose gels in TAE buffer. A 100 base pair DNA ruler loaded in the first lane served as length standard. B) Distribution pattern for UCHL1 promoter DNA methylation in tumor adjacent kidney epithelium, autologous primary RCC lesions and RCC cell lines. Grey bars represent samples with unmethylated (U), striped bars with partially methylated (P) and black bars with fully methylated (M) CpG islets within the UCHL1 promoter core region as indicated.

Since an impaired UCHL1 expression was not only found in RCC cell lines, but also at a high frequency in primary RCC lesions [21], the methylation status of the UCHL1 promoter DNA in 32 biopsy systems each comprised of a primary RCC lesions as well as corresponding non-neoplastic tumor adjacent kidney epithelium tissues was determined. As representatively shown in Figure 3A, COBRA analysis revealed partial UCHL1 promoter DNA methylation in the RCC lesions 2874 and 2876, whereas the lesion 2878 represented a tumor with a largely demethylated UCHL1 promoter DNA region. In contrast to the COBRA pattern characteristic for partial or rare promoter DNA methylation MZ1940RC cells represent a COBRA pattern characteristic for total promoter DNA methylation. Overall, the COBRA analyses revealed that 12/32 primary RCC lesions could be classified as partially methylated in regard to their UCHL1 promoter, whereas no methylation was found in the tumor adjacent kidney epithelium. The methylation status of RCC lesions was comparable to that of RCC cell lines, in which 9/17 RCC cell lines lack methylation, 3/17 exhibit a partial and 5/17 a total UCHL1 promoter DNA methylation (Table 1). In addition the sequencing of bisulfite-treated DNA confirmed the distinct methylation status of the UCHL1 promoter in the RCC lesions (data not shown). Thus, the epigenetic inactivation of UCHL1 is a common event in both primary RCC cell lines and RCC lesions and may represent a mechanism for its functional loss observed in the early phase of this disease.

## Discussion

Promoter DNA methylation has been associated with the regulation of the expression pattern of tumor markers defined in both primary tumor specimen as well as in body fluids [40-42]. In RCC aberrant DNA methylation of the tumor suppressor gene VHL is found at a high frequency, whereas the frequencies of DNA promoter methylation of other tumor suppressor genes vary in this malignancy [22,43].

UCHL1, an essential member of the proteasome targeting ubiquitin-dependent protein degradation pathway plays an important role in distinct cellular processes such as cell proliferation, cell cycle, apoptosis and intracellular signalling [8], which are often disturbed in cancers [44,45]. UCHL1 has been demonstrated to be either overexpressed or silenced in both tumor lesions and/or tumor cell lines of distinct origin [12-14,21,46]. UCHL1 overexpression as found in colorectal cancer, non-small cell lung carcinoma and RCC was associated with a more aggressive potential and/or metastatic phenotype as well as in some cases with a poor prognosis of the respective patient collectives [17,19,21,47]. In contrast, UCHL1 expression has been also shown to be associated with increased apoptosis in breast cancer cells [48]. However, these studies did not analyse the underlying molecular mechanism of the heterogeneity of UCHL1 expression levels. The silencing of UCHL1 was discovered by cDNA microarrays and chemical genomic screening of head and neck squamous cell carcinoma [49] as well as pancreatic carcinoma lesions and pancreatic carcinoma cells either left untreated or treated with demethylating agents [32,35]. In addition, the silencing or downregulation of UCHL1 mediated by hypermethylation in esophageal squamous cell, hepatocellular and gallbladder carcinoma was correlated in these diseases with a poor prognosis of patients [14,31,50]. However, there exist some discrepancies in terms of the existing UCHL1 promoter methylation status, which might at least partially explained by the different methods employed for determination of the promoter DNA methylation status. In our hands, direct bisulfite sequencing is the most sensitive method when compared to methylation-specific PCR and/or COBRA analyses and has the further advantage of allowing the quantification of the methylation/demethylation ratio.

Beside DNA methylation there exist other gene silencing mechanisms, such as the modification of the histone structure by inappropriate deacetylation, or the presence of the recently discovered microRNAs, which can either act as selective destructors of targeted mRNA transcripts or block the translation of mRNAs.

However, in this study it is demonstrated that the silencing of UCHL1 in both RCC cell lines as well as in primary RCC lesions mostly of clear cell subtype is rather linked to the methylation of the UCHL1 promoter DNA. This is further supported by the fact that a correlation between the methylation status of the CpG islet in the UCHL1 promoter DNA and the expression pattern at the transcriptional as well as the translational level is shown. Since UCHL1 protein expression is more pronounced in metastatic than in primary RCC lesions [20], one can speculate that UCHL1 expression is actively silenced during the early stages of tumorigenesis and that its restored expression at a later stage may rather represent a reliable marker for metastatic disease. This is in accordance with a recent paper demonstrating a high frequency of UCHL1 methylation in primary RCC when compared to normal kidney epithelium [36]. Similar results were obtained in colorectal cancer demonstrating a lower frequency of methylation in metastasis when compared to the primary tumor [51]. However, the methylation pattern of UCHL1 might not only serve as a prognostic and/or predictive marker and reflect the metastatic potential of RCC, but might also modulate the therapy sensitivity thereby influencing the treatment modalities of RCC patients.

The function of UCHL1 in tumors is still controversially discussed. In some tumor entities a hypermethylation of UCHL1 was demonstrated in the primary tumor suggesting a tumor suppressor gene activity, whereas in other tumor types UCHL1 was highly overexpressed as a cause/consequence of the transformation process. The initial downregulation of UCHL1 by DNA promoter methylation might provide a growth advantage for these tumor cells and thus represent a tumor escape mechanism since the antigen cannot be recognized by the immune system [34]. However, the functional consequences of temporary UCHL1 inactivation still need to be determined. In the UCHL1 knock out mice (gad mice) ubiquitin levels were not induced and did not modulate the apoptosis-sensitive phenotype [8]. If changes in the methylation pattern are involved in the development of resistance against chemotherapy and radiation in cancer cells, the determination of the given methylation status of the UCHL1 promoter may contribute to the understanding of the role of a differential UCHL1 expression during tumorigenesis and progression of human cancers as well as in the course of developing therapy resistance. UCHL1 is characterized by its dual function as a hydrolase in order to generate free ubiquitins and as a ligase involved in producing multi-ubiquitinated proteins [52]. The reexpression of UCHL1 in metastatic RCC indicated a tumor stage-specific UCHL1 hypomethylation suggesting that UCHL1 acts as an oncogene rather than as a tumor suppressor gene. However, it still has to be defined, which proteins might be protected from (UCHL1 deubiquitination activity) or alternatively directed to undergo (UCHL1 ubiquitin ligation activity) proteasomal degradation. Possible candidates for its rescue activity might be proteins contributing to the chemo- and radiation resistance of RCC such as multi drug resistance factors, whereas the targeted degradation of apoptosis inducing factors might help to evade such elimination mechanisms.

Since UCHL1 (over)expression frequently occurs during tumor progression this protein might be beneficial for the progression and metastases formation process in certain cancers [12,19,21]. This concept is further strengthened by an enhanced cell proliferation and migration capacity observed upon UCHL1 overexpression in UCHL1^- ^RCC cell lines [21].

In addition it has been shown that UCHL1 interacts with the jun activating binding protein JAB1 and p27^Kip ^[53]. Due to the interaction with JAB1, p27^Kip ^is degraded in the cytoplasm leading to reduced p27^Kip ^expression levels. However, a relationship between UCHL1 and p27^Kip ^expression in cancers including RCC has also not yet been determined.

Promoter DNA methylation has been linked to the expression of tumor markers not only defined in primary tumors, but also in body fluids [40-42,54]. Indeed, cancer-specific DNA methylation pattern can be detected in circulating tumor cells of the body fluids, such as urine and blood. If UCHL1 methylation is RCC-related, detection of UCHL1 DNA promoter methylation in addition to the existence of UCHL1-specific autoantibodies detected in sera of tumor patients [46,55,56] may further help to define patients with poor prognosis. Thus one upcoming aim that will be addressed in the near future is to determine the suitability of UCHL1 as a serum marker in order to distinguish between patients with different clinical outcome.

## List of abbreviations

ab: antibody; COBRA: combined bisulfite restriction analysis; DAC: 2'-deoxy-5-azacytidine; DMSO: dimethylsulfoxide; DUB: deubiquinating enzymes; FCS: fetal calf serum; HRP: horseradish peroxidase; PCR: polymerase chain reaction; PGP: protein gene product; RCC: renal cell carcinoma; RT: reverse transcription; UCH: ubiquitin carboxyl-terminal hydrolases; UCHL1: ubiquitin carboxyl-terminal hydrolase 1; USP: ubiquitin-specific proteases; VHL: von Hippel Lindau.

## Competing interests

The authors declare that they have no competing interests.

## Authors' contributions

BS: idea, experimental design, manuscript preparation, data interpretation. DH: experiments, methylation studies. ES: experiments, mRNA and protein expression. JB: primer design, data analyses and interpretation. RL: manuscript preparation, data interpretation. RD: experimental design, methylation studies.

## Supplementary Material

Additional file 1 **Schematic view of the UCHL1 promoter DNA methylation status in representative renal tissue samples and RCC cell lines based on bisulfite sequencing data.**  A) Representative UCHL1 promoter DNA methylation status of the biopsy system MZ2874. Three independent sequences derived from the UCHL1 promoter-specific amplicons representing either tumor adjacent renal tissue or the RCC tumor lesion were subjected to bisulfite sequencing. Genomic DNA was extracted from the distinct samples treated with bisulfite amplified by nested PCR and subsequently subjected to sequencing as described in the Methods section. The 3 upper lanes show the methylation status of the UCHL1 core promoter region in 3 independent sequences representing tumor adjacent renal tissue (NN) whereas the 3 lower lanes the methylation status as defined in three independent tumor sequences (TU). The 22 circles shown in each lane correspond to the schematic view of the UCHL1 promoter DNA region shown in Figure 1A. Open circles represent unmethylated CpG sites whereas methylated sites are indicated by black circles. B) Representative UCHL1 promoter methylation pattern of RCC cell lines. The 2 upper lanes (MZ1257RC and MZ1851RC) represent examples for RCC cell lines with unmethylated UCHL1 promoter DNA regions (U), the 2 middle lanes (MZ2862RC and MZ2904) for RCC cell lines with partially methylated UCHL1 promoter DNA regions and the 2 lower lanes for RCC cell lines (MZ1851LN and MZ1940RC) with fully methylated UCHL1 promoter DNA regions. Sample handling as well as the layout are in analogy to Additional file 1A. CpG sites for which the methylation status could not be defined are indicated by gaps. Click here for file
