# Supplementary material for: Epigenetic control of the ubiquitin carboxyl terminal hydrolase 1 in renal cell carcinoma
Source: J Transl Med. 2009 Oct 26;7:90. doi: 10.1186/1479-5876-7-90 (PMC2775027; doi:10.1186/1479-5876-7-90)
Supplement: Additional file 1 — Schematic view of the UCHL1 promoter DNA methylation status in representative renal tissue samples and RCC cell lines based on bisulfite sequencing data. A) Representative UCHL1 promoter DNA methylation status of the biopsy system MZ2874. Three independent sequences derived from the UCHL1 promoter-specific amplicons representing either tumor adjacent renal tissue or the RCC tumor lesion were subjected to bisulfite sequencing. Genomic DNA was extracted from the distinct samples treated with bisulfite amplified by nested PCR and subsequently subjected to sequencing as described in the Methods section. The 3 upper lanes show the methylation status of the UCHL1 core promoter region in 3 independent sequences representing tumor adjacent renal tissue (NN) whereas the 3 lower lanes the methylation status as defined in three independent tumor sequences (TU). The 22 circles shown in each lane correspond to the schematic view of the UCHL1 promoter DNA region shown in Figure 1A. Open circles represent unmethylated CpG sites whereas methylated sites are indicated by black circles. B) Representative UCHL1 promoter methylation pattern of RCC cell lines. The 2 upper lanes (MZ1257RC and MZ1851RC) represent examples for RCC cell lines with unmethylated UCHL1 promoter DNA regions (U), the 2 middle lanes (MZ2862RC and MZ2904) for RCC cell lines with partially methylated UCHL1 promoter DNA regions and the 2 lower lanes for RCC cell lines (MZ1851LN and MZ1940RC) with fully methylated UCHL1 promoter DNA regions. Sample handling as well as the layout are in analogy to Additional file 1A. CpG sites for which the methylation status could not be defined are indicated by gaps. [file 1479-5876-7-90-S1.PPT]

## Slide 1
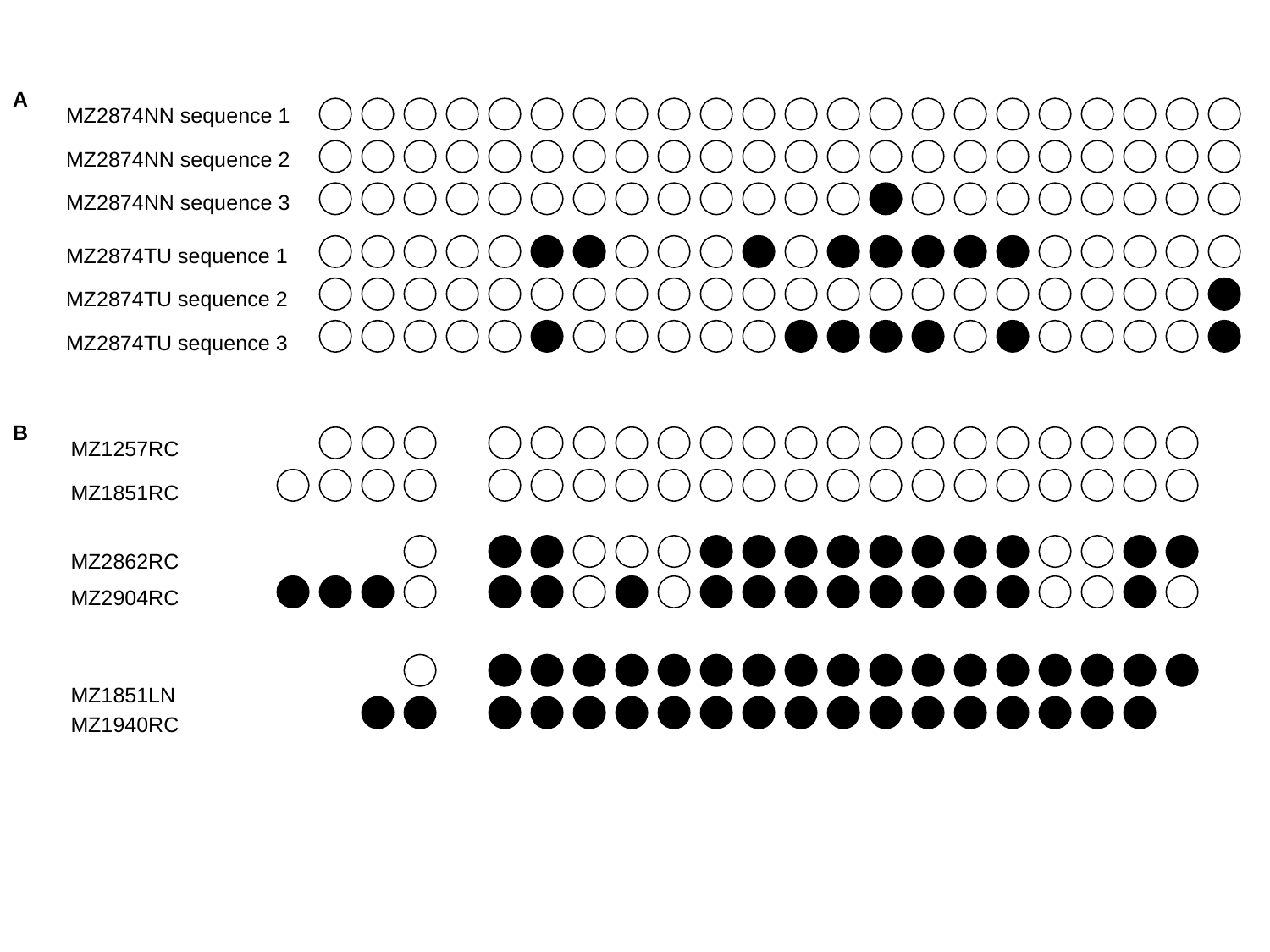

A
MZ2874NN sequence 1
MZ2874NN sequence 2
MZ2874NN sequence 3
MZ2874TU sequence 1
MZ2874TU sequence 2
MZ2874TU sequence 3
B
MZ1257RC
MZ1851RC
MZ2862RC
MZ2904RC
MZ1851LN
MZ1940RC
